# Smartphone motor testing to distinguish idiopathic REM sleep behavior disorder, controls, and PD

**DOI:** 10.1212/WNL.0000000000006366

**Published:** 2018-10-16

**Authors:** Siddharth Arora, Fahd Baig, Christine Lo, Thomas R. Barber, Michael A. Lawton, Andong Zhan, Michal Rolinski, Claudio Ruffmann, Johannes C. Klein, Jane Rumbold, Amandine Louvel, Zenobia Zaiwalla, Graham Lennox, Tim Quinnell, Gary Dennis, Richard Wade-Martins, Yoav Ben-Shlomo, Max A. Little, Michele T. Hu

**Affiliations:** From the Oxford Parkinson's Disease Centre (OPDC) (S.A., F.B., C.L., T.R.B., M.R., C.R., J.C.K., J.R., A.L., R.W.-M, M.T.H.), University of Oxford, UK; Engineering and Applied Science (S.A., M.A.L.), Aston University, Birmingham, UK; Somerville College (S.A.), University of Oxford, UK; Nuffield Department of Clinical Neurosciences (F.B., C.L., T.R.B., M.A.L., M.T.H.), University of Oxford, UK; Population Health Sciences (M.A.L.), University of Bristol, UK; andDepartment of Computer Science (A.Z.), Johns Hopkins University, Baltimore; Department of Neurology and Neurophysiology (Z.Z., G.L., M.T.H.), Oxford University Hospitals NHS Trust, UK; Respiratory Support and Sleep Centre (T.Q.), Papworth Hospital, Cambridge, UK; Department of Neurology (G.D.), Royal Hallamshire Hospital, Sheffield, UK; and Media Lab (M.A.L.), Massachusetts Institute of Technology, Cambridge, MA.

## Abstract

**Objective:**

We sought to identify motor features that would allow the delineation of individuals with sleep study-confirmed idiopathic REM sleep behavior disorder (iRBD) from controls and Parkinson disease (PD) using a customized smartphone application.

**Methods:**

A total of 334 PD, 104 iRBD, and 84 control participants performed 7 tasks to evaluate voice, balance, gait, finger tapping, reaction time, rest tremor, and postural tremor. Smartphone recordings were collected both in clinic and at home under noncontrolled conditions over several days. All participants underwent detailed parallel in-clinic assessments. Using only the smartphone sensor recordings, we sought to (1) discriminate whether the participant had iRBD or PD and (2) identify which of the above 7 motor tasks were most salient in distinguishing groups.

**Results:**

Statistically significant differences based on these 7 tasks were observed between the 3 groups. For the 3 pairwise discriminatory comparisons, (1) controls vs iRBD, (2) controls vs PD, and (3) iRBD vs PD, the mean sensitivity and specificity values ranged from 84.6% to 91.9%. Postural tremor, rest tremor, and voice were the most discriminatory tasks overall, whereas the reaction time was least discriminatory.

**Conclusions:**

Prodromal forms of PD include the sleep disorder iRBD, where subtle motor impairment can be detected using clinician-based rating scales (e.g., Unified Parkinson's Disease Rating Scale), which may lack the sensitivity to detect and track granular change. Consumer grade smartphones can be used to accurately separate not only iRBD from controls but also iRBD from PD participants, providing a growing consensus for the utility of digital biomarkers in early and prodromal PD.

Polysomnographically confirmed idiopathic REM sleep behavior disorder (iRBD) is associated with rates of phenoconversion to a neurodegenerative disorder, most often a synucleinopathy, of up to 91% over a 14-year follow-up period.^1–3^ Such individuals therefore offer an enriched population in which to study potential neuroprotective treatments. In otherwise asymptomatic individuals with iRBD, the detection of subtle motor impairment may portend relatively imminent conversion to Parkinson disease (PD).^4^ A major challenge is the availability of robust outcome measures, resistant to inherent intra- and inter-rater differences associated with physician-rated scales and placebo/nocebo treatment effects, that can sensitively measure short-term progression.^5,6^

With advances in technology has come the hope of delivering objective measures of disease severity, with multiple measures permitting the tracking of symptoms over time.^7^ Several devices have garnered popularity. Differences in their interfaces and calculated measures belie the commonality of their hardware, which typically comprise integrated accelerometers and gyroscopes measuring motor impairment.^8^ Such inertial measurement units (IMUs) are also integrated into consumer grade smartphones, increasing in ubiquity worldwide.

We have previously evaluated the feasibility and efficacy of smartphone use in detecting and monitoring the symptoms of PD in a pilot study, assessing voice, balance, gait, finger tapping, and reaction time.^9^ We now investigate the larger scale use of smartphones under more realistic clinic- and home-based conditions to objectively quantify motor symptoms in the deeply phenotyped Oxford Discovery cohort.^4,10^ Here, our aims were to (1) distinguish participants with iRBD from controls and PD and (2) identify the most salient motor features that distinguish between groups.

## Methods

### Participant selection

Data were collected from participants enrolled in the Oxford Parkinson's Disease Centre (OPDC) Discovery study^4,10^ using smartphone assessments at their clinic visit and then at home over a maximum of 7 days. A diagnosis of iRBD was made by a sleep specialist, supported by polysomnography, concordant with the American Academy of Sleep Medicine International Classification of Sleep Disorders criteria.^11^ Individuals with idiopathic PD had a high clinician determined probability (≥90%) of PD, confirmed on their most recent longitudinal assessment.

### Standard protocol approvals, registrations, and patient consents

The study protocol was approved by the local UK National Health Service Ethics committee, in adherence with national legislation and the Declaration of Helsinki. All participants provided written informed consent at the point of recruitment.

### Smartphone test protocol

**Figure 1:**
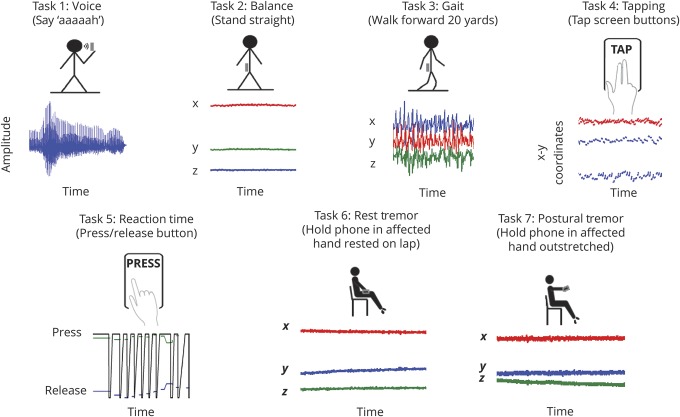
Schematic diagram illustrating the major steps involved in data acquisition of 7 smartphone tasks assessing voice, balance, gait, finger tapping, reaction time, rest tremor, and postural tremor For the voice task, using the inbuilt microphone, we recorded the sustained phonation “aaah”; the participants were instructed to “Hold the phone to your ear, take a deep breath, and say “aaah” at a comfortable and steady, tone and level, for as long as you can.” For the balance task, using the smartphone inertial measurement units (IMUs), we collected triaxial accelerometer sensor data; the participants were instructed to “Stand up straight and place the phone in your pocket. When the buzzer vibrates, stay standing until the buzzer vibrates again.” For the gait task, using the smartphone IMUs, we collected triaxial accelerometer sensor data; the participants were instructed to “Stand up and place the phone in your pocket. When the buzzer vibrates, walk forward 20 yards. Then, stop, turn around, and walk back again.” For the finger tapping task, using the touch screen sensors and timer, we recorded time and location (*x-y* screen coordinate position) of finger touch; the participants were instructed to “Tap the buttons below with the index and middle fingers of 1 hand alternatively, in a regular rhythm.” For the reaction time task, using the touch screen sensors and timer, we recorded the time of stimulus onset (appearance/disappearance of a screen button) and response (press/release the screen button) along with location (*x-y* screen coordinate position) of finger touch; the participants were instructed to “Press the screen button below as soon as it appears; release as soon as it disappears.” For the rest tremor task, using the smartphone IMUs, we collected triaxial accelerometer sensor data; the participants were instructed to “Sit upright, hold the phone in your tremor dominant hand and rest it lightly in your lap, and close your eyes and count backward from 100.” For the postural tremor task, using the smartphone IMUs, we collected triaxial accelerometer sensor data; the participants were instructed to “Sit upright and hold the phone in your tremor dominant hand, with the arm outstretched in front of you.”

Details regarding the smartphone test protocol used in this study have been described previously.^9^ This prompted participants to perform 5 short tasks (less than 5 minutes overall) to assess: (1) voice, (2) balance, (3) gait, (4) finger tapping, and (5) reaction time. The smartphone application was adapted to utilize integrated smartphone IMUs^12^ to allow 2 additional tasks for tremor (about 45 seconds each) assessing (6) rest tremor, instructing the user to “sit upright, hold the phone in your tremor dominant hand and rest it lightly in your lap, and close your eyes and count backward from 100,” and (7) postural tremor, instructing the user to “sit upright and hold the phone in your tremor dominant hand, with the arm outstretched in front of you” (figure 1). IMU sensor data were encrypted, timestamped, and uploaded to a secure online database.

### Data preprocessing

Previous studies on objective PD symptom detection have typically relied on high-quality sensor data collected in a controlled laboratory environment using expensive hardware (e.g., a double-walled sound booth^13,14^ and a gait laboratory with cameras and forceplates^15^). The collection of sensor recordings under more realistic conditions outside the laboratory, using consumer-grade smartphones, potentially makes the protocol in this study practical and scalable for clinical practice. However, it also results in uncontrolled factors that may affect the sensor data. To identify and distinguish useful from artifactual segments of sensor data, we used an automated segmentation algorithm. Smartphone recordings were included only if all 7 tasks were performed in succession, with sufficiently similar timestamps. Synchronization of data based on timestamps allows for the combination of information from different sensors, thereby facilitating simultaneous analyses of all 7 smartphone tasks.

### Feature extraction

Voice impairments in PD are typically characterized by roughness, breathiness, and exaggerated vocal tremor.^16^ Recently, speech abnormalities have also been demonstrated in iRBD compared with control participants (sensitivity, 96%; specificity, 79%).^17,18^ We calculated a range of features using the sustained phonation “aaah” (International phonetic alphabet /a:/) based on previous work data available from Dryad (table e-1, doi.org/10.5061/dryad.3qm0152).^13,14,19^

Deficits in repetitive finger tapping tasks in PD include hastening, faltering, or freezing.^20^ Using the screen pixel position (*x-y* coordinates) and the timing of touch, we extracted 2 categories of summary measures; temporal features and spatial features data available from Dryad (table e-2, doi.org/10.5061/dryad.3qm0152).

There is no general consensus regarding the existence and nature of reaction time deficits in PD.^21^ A selective deficit in simple reaction time compared with choice reaction time has been suggested, but findings are dependent on experimental conditions.^21,22^ We analyzed noncued simple reaction time, using the elapsed time between the stimulus (appearance/disappearance of a screen button) and response (press/release of the button), to extract features based on the descriptive properties of the reaction time data available from Dryad (table e-3, doi.org/10.5061/dryad.3qm0152).

Gait and balance deficits in PD are typically characterized by episodes of freezing of gait, falling, shuffling, progressive loss of postural reflexes, and festination.^23^ A recent study demonstrated that rest and postural tremor can be used to discriminate PD from controls and PD from essential tremor.^12^ In this study, for the 4 IMU-based tasks, namely balance, gait, rest tremor, and postural tremor, we extracted 5 categories of summary measures data available from Dryad (table e-4, doi.org/10.5061/dryad.3qm0152).

### Feature selection

Identification of the most salient features having the highest discriminatory power was undertaken using different feature selection algorithms. Feature selection enhances the explanatory power of the analysis by removing redundant and less informative features, which helps reduce the complexity of the discriminatory analysis. Five different feature selection algorithms were used. This resulted in 5 different rankings, 1 from each algorithm. Majority voting was used to derive a single unified ranking to be used for inference and discrimination.^24^ Combining outputs from different algorithms can improve the reliability of feature rankings, as multiple algorithms tend to reduce the potential variability associated with using an individual technique.^25^

### Statistical analysis

Three pairwise discriminatory comparisons were considered: (1) controls vs iRBD, (2) controls vs PD, and (3) iRBD vs PD. Statistical model predictions were compared with clinicians' assessments, which were treated as the ground truth. Statistical analysis was aimed at (1) objective quantification of motor symptoms using the sensor data, (2) identification of the most salient features that help discriminate the 3 groups, and (3) assessment of the discrimination accuracy (sensitivity and specificity) for the respective pairwise comparisons. To assess and quantify motor symptoms, we extracted a range of summary measures (features) to characterize the symptom-relevant properties of the sensor data. Contrasting with previous studies that have typically focused on assessing 1 motor symptom in PD, statistical analyses in this study were based on the acquisition, quantification, and analysis of 7 different motor characteristics.

### Internal validation

To discriminate the 3 contrast groups, we used a statistical machine learning method (random forests), which is commonly used to separate generic data into several classes.^26^ To validate the method, we used randomized cross-validation (CV). CV helps assess the generalizability of a model to similar previously unseen data sets. CV involves repetitive splitting of the data into nonoverlapping “training” and “validation” sets. The training data are used to find discriminatory patterns in the features. The validation set is used to assess the method's discrimination accuracy; effectively, the random forest classifier is blinded to these data during training.

We used 3 different CV methods: (1) 10-fold CV, (2) leave-one-subject-out (LOSO), and (3) leave-one-(recording)-out (LOO). Validation based on 10-fold CV has been commonly used in other studies.^13,14,19^ The data are split such that 90% of randomly selected recordings are used for training, whereas the remaining 10% are used for validation. LOSO CV involves splitting the data such that all recordings from only 1 randomly selected participant are used in the validation set, whereas recordings from all remaining participants are used for training. LOO CV uses 1 randomly selected recording for validation, whereas all remaining recordings are used for training. A single recording (comprising each of the 7 tasks) was used for each participant. To account for differences in group sample sizes, an equal number of recordings were randomly selected before training and validation from the groups being compared at each CV repetition. Data available from Dryad (additional methods, doi.org/10.5061/dryad.3qm0152) show further details on feature extraction, feature selection, data imputation, and CV.

To gauge the association between the accuracy and number of features, validation was undertaken using different numbers of the most salient features. Accuracy was quantified using sensitivity and specificity. Discrimination accuracies were computed separately at each CV iteration and summarized using mean and SD. To account for potential differences in sex, sensitivity and specificity were computed separately for all recordings, only female recordings, and only male recordings.

**Figure 2:**
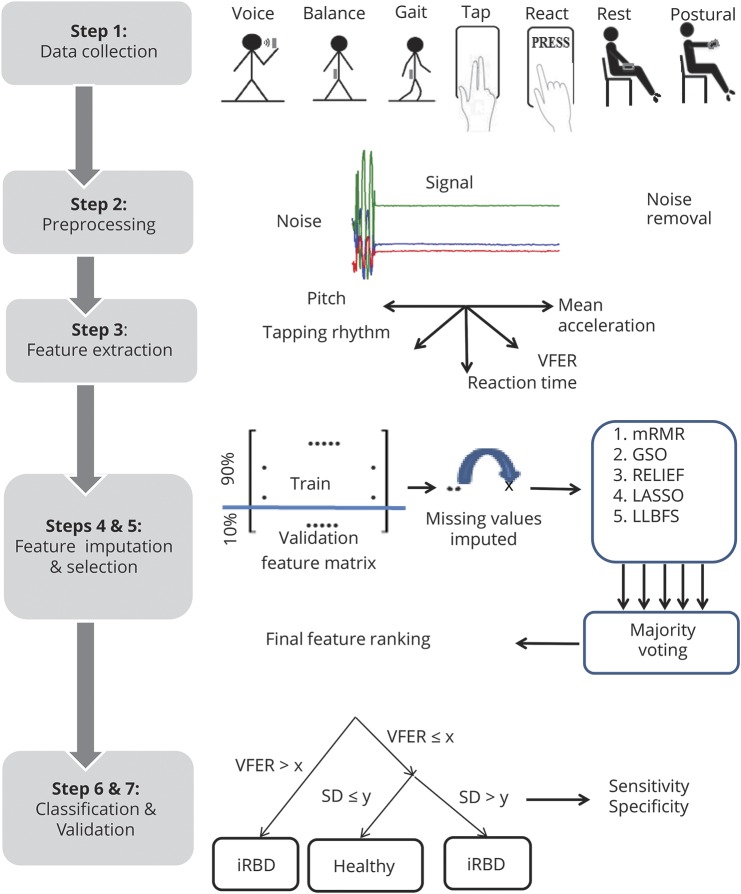
Schematic diagram illustrating the major steps involved in the analysis of smartphone sensor data from 7 smartphone tasks assessing voice, balance, gait, finger tapping, reaction time, rest tremor, and postural tremor in the smartphone app used in this study GSO = Gram-Schmidt orthogonalization; LASSO = least absolute shrinkage and selection operator; LLBFS = local learning-based feature selection; mRMR = minimum redundancy maximum relevance; PD = Parkinson disease; iRBD = idiopathic REM sleep behavior disorder; VFER = vocal fold excitation ratio.

Significance was set at 5% (unless otherwise stated), and hypothesis tests were 2 sided. Statistical analysis was performed using Matlab software (version 2016b). The overall analysis steps are illustrated in figure 2.

### Data availability

The OPDC have a Data Access Committee whose function is to promote scientific collaboration and maximize the benefit of our research for the wider community. Individual deidentified participant data can be made available via a formal application process to the OPDC Data Access Committee by any qualified investigator, as outlined in our web site: opdc.medsci.ox.ac.uk/external-collaborations, which contains the application form, protocol, and terms and conditions.

## Results

The demographic characteristics of the participants are shown in table 1. As expected, iRBD participants were younger and more likely to be men. Other clinical differences were evident, associated with disease status. Control, iRBD, and PD participants contributed on average 9.5, 13.1, and 8.2 recordings (each comprising all 7 tasks), respectively. For each of the 7 tasks, this resulted in a total of 799 control, 1,358 iRBD, and 2,734 PD recordings (table 2). From the 7 tasks, a total of 998 summary features were extracted. Using all 998 features, the mean sensitivity and specificity values ranged from 84.6% (SD 4.1%) to 91.9% (SD 3.5%) and 88.3% (SD 3.3%) to 90.1% (SD 2.7%), respectively, for all 3 pairwise comparisons (table 2).

**Table 1:**
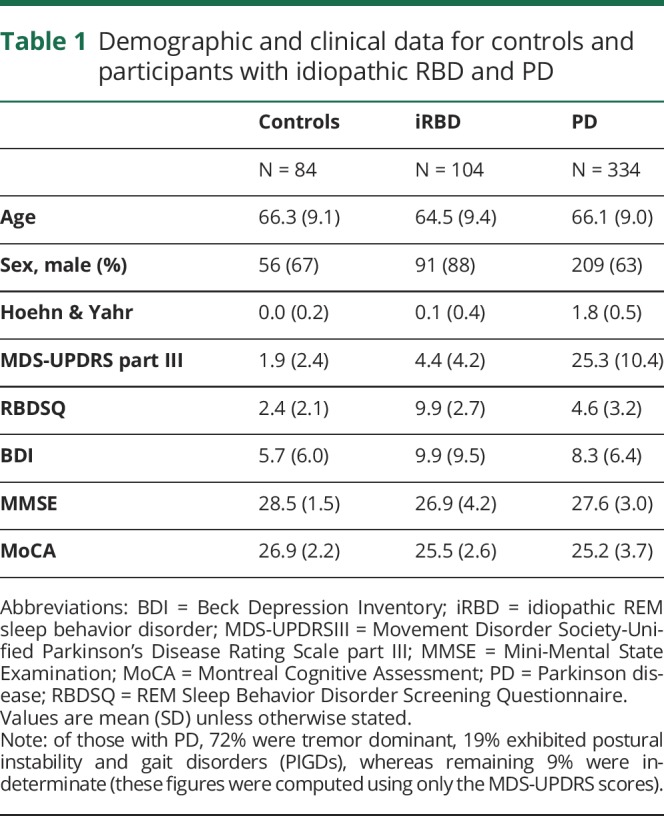
Demographic and clinical data for controls and participants with idiopathic RBD and PD

**Table 2:**
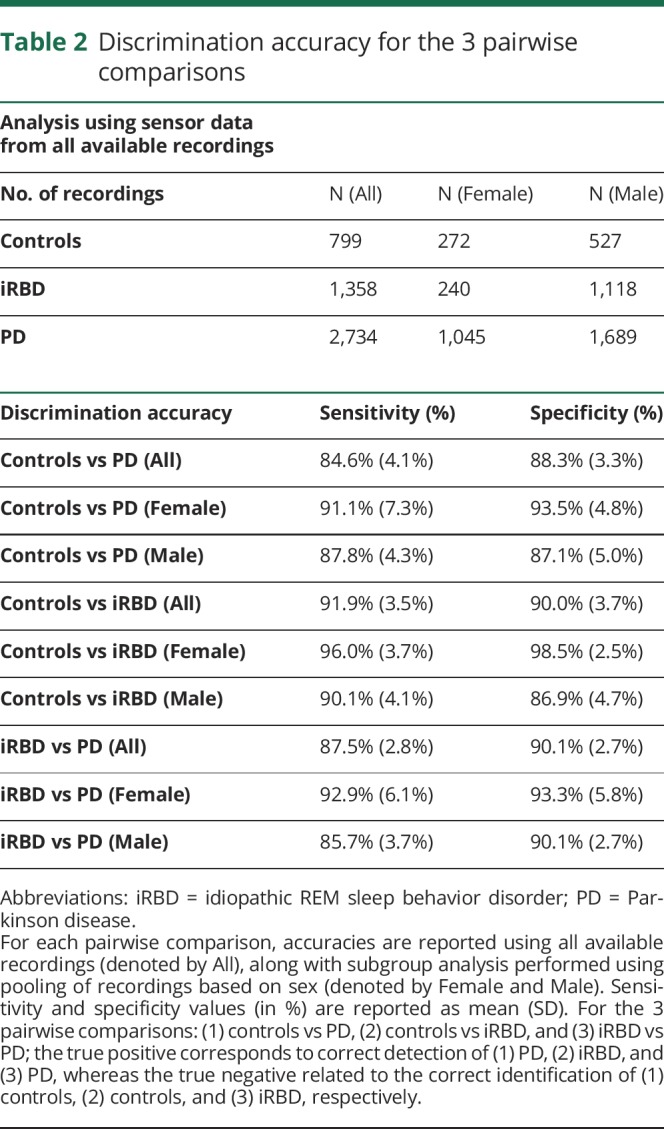
Discrimination accuracy for the 3 pairwise comparisons

**Figure 3:**
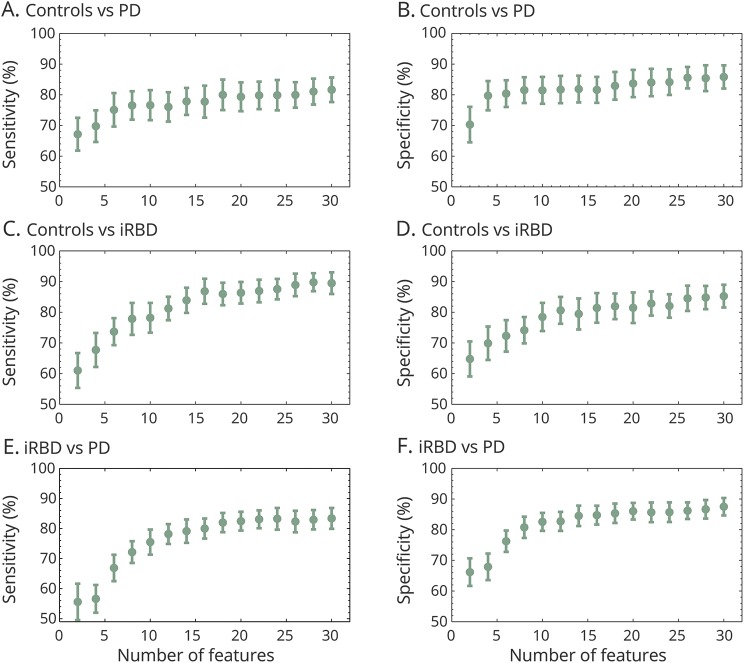
Discrimination accuracies as a function of the number of salient features used in the machine learning discrimination analysis, for the 3 pairwise comparisons: controls vs Parkinson disease (PD) (panels A and B), controls vs idiopathic REM sleep behavior disorder (iRBD) (panels C and D), and iRBD vs PD (panels E and F) The above accuracies were computed using all available recordings from the 3 clinical groups, using all 998 features computed from the 7 tasks, using 10-fold cross-validation (10 repetitions). The rankings of the most salient features were obtained using a majority voting scheme (using 5 feature selection algorithms). The feature rankings were obtained separately for each of the above 3 pairwise comparisons. Features were added into the machine learning classifier (random forest) in increments of 2 (starting from 2 and going up to 30), whereby higher ranked features were added first. The whole process of training and validation was repeated each time 2 new features were included. Sensitivity and specificity values (in %) were reported as mean (denoted by gray circles) and SD (vertical bars).

As expected, the sensitivity and specificity values increased as more features were incorporated (figure 3). Accuracies obtained using the 30 most salient features were broadly comparable with the corresponding sensitivity and specificity values obtained using all 998 features. The increase in discrimination accuracy for sex-specific subgroup analyses is provided in data available from Dryad (figures e-1 and e-2, doi.org/10.5061/dryad.3qm0152), which show that the sex-specific results are in overall agreement with accuracies obtained using all available recordings (figure 3).

Using only the 30 top-ranked features, the mean sensitivity and mean specificity was (1) 89.5% (SD 3.5%) and 85.3% (SD 3.7%) in discriminating controls from iRBD, (2) 83.4% (SD 3.5%) and 87.5% (SD 2.8%) in discriminating iRBD from PD, and (3) 81.7% (SD 4.0%) and 85.8% (SD 3.8%) in discriminating PD recordings from controls, where male and female participants were combined across groups. All sensitivity and specificity results were significantly better than comparable results obtained using completely randomized predictions (*p* < 0.001, 2-sided Kolmogorov-Smirnov test). See data available from Dryad (tables e-5 to e-7, doi.org/10.5061/dryad.3qm0152) for details regarding the most salient features.

Using only a single recording (comprising all 7 tasks that were performed for the very first time) for each participant, the LOO CV accuracy in discriminating PD participants from controls was slightly higher compared with the other 2 group comparisons (controls vs iRBD and iRBD vs PD; data available from Dryad, table e-8, doi.org/10.5061/dryad.3qm0152). Using only the 30 most salient features for each pairwise comparison, the mean discrimination accuracy using the LOSO CV scheme (using all recordings by a given participant over time) for the 3 pairwise comparisons was around 70%–75% (data available from Dryad, table e-9, doi.org/10.5061/dryad.3qm0152). The accuracies obtained using all recordings for the 3 validation schemes were significantly better than comparable results obtained using completely randomized predictions. Percentage mismatch in the features of training and validation datasets using the 10-fold CV and LOSO CV schemes is also provided (data available from Dryad, figures e-3 and e-4, doi.org/10.5061/dryad.3qm0152); unfortunately, LOSO CV mismatch was too large for this form of validation to be considered statistically reliable.

**Figure 4:**
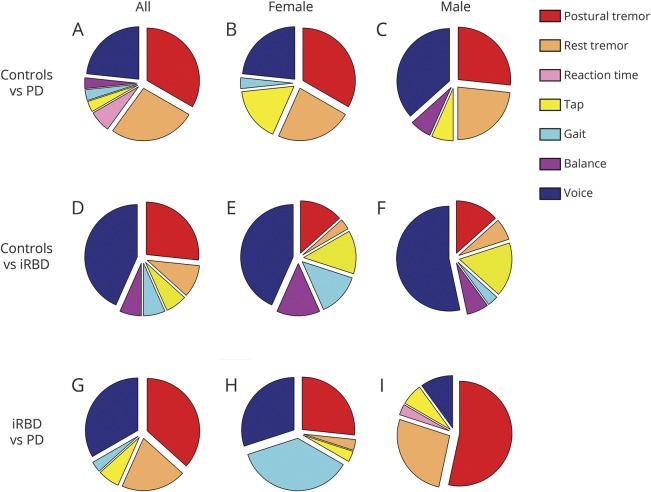
Graphical illustration of the most salient discriminatory tasks for the 3 pairwise comparisons: controls vs Parkinson disease (PD) (charts A–C, top horizontal panel), controls vs idiopathic REM sleep behavior disorder (iRBD) (charts D–F, middle horizontal panel), and iRBD vs PD (charts G–I, lower horizontal panel) The above pie charts were generated using the 30 most salient features computed from the smartphone sensor recordings. The rankings of the most discriminatory features were obtained using a majority voting scheme (using 5 feature selection algorithms). The feature rankings were obtained separately for each of the above 9 pairwise comparisons. For a given pairwise comparison, charts were generated by computing the percentage of features that were selected for each of the 7 smartphone tasks. A larger pie segment corresponds to smartphone tasks that were identified as being relatively more discriminatory for the pairwise comparison under consideration. For each comparison, task rankings were computed using all available recordings (denoted by All, leftmost vertical panel), along with a subgroup analysis performed using pooling of observations for females and males, denoted by Female (middle vertical panel) and Male (rightmost vertical panel), respectively.

Voice was the most discriminatory factor between iRBD and controls, by sex and overall, constituting approximately 50% of the most salient features (figure 4, D–F). A preponderance of gait-related features is evident in female iRBD participants, but conclusions that may be drawn are limited by the small numbers recruited.

Postural and rest tremor were the second most salient factors in discriminating iRBD and controls. Features derived from tasks assessing tremor were the major discriminant between PD and controls and PD and iRBD, accounting for 60% and 57% of the 30 top features, respectively (figure 4, A and G). This is broadly consistent with motor phenotyping in other early PD cohorts using the Movement Disorders Society-Unified Parkinson's Disease Rating Scale (MDS-UPDRS) to separate into motor phenotype,^27,28^ revealing 72% with tremor-dominant PD (this figure was calculated using only the MDS-UPDRS scores; for details, see Stebbins et al.^28^). Voice was the third most discriminatory factor between PD and controls and the second most salient distinguishing task between iRBD and PD. Reflected in MDS-UPDRS III, individuals with iRBD and controls were more similar to each other than PD in terms of motor symptoms. As expected, task rankings for the comparisons of PD vs controls and PD vs iRBD were also similarly comparable (figure 4).

Across different pairwise comparisons, noncued reaction time was one of the least useful tasks overall in discriminating between groups, in keeping with previous reports of significant deficits in reaction time among PD participants under cued but not noncued conditions.^29^ In-clinic observations noticed that participants had different levels of engagement with the reaction time task, with some finding it challenging to perform the task as per the instructions at the start.

## Discussion

We report the results of smartphone assessments in one of the largest cohorts of deeply phenotyped participants with iRBD, PD and controls, demonstrating that objective quantification of motor symptoms using smartphones can be used to discriminate between participant groups with a high level of accuracy (84.6%–91.9% mean sensitivity and specificity). We were able to generate a comparable level of accuracy using the top-ranked 30 of the total 998 derived features. The performance characteristics were similar for men and women. Perhaps surprisingly, these smartphone sensor data performed equally as well for the iRBD to control comparison as it did for the PD to control comparison, although clinically PD participants generally have far more pronounced motor features than prodromal iRBD participants.

Of note, after voice, we found that postural and rest tremor were salient factors in discriminating iRBD from controls. This may be surprising to many sleep specialists; however, mild or intermittent postural tremor signs and symptoms may not be clinically evident during outpatient review and infrequent MDS-UPDRS rating. Moreover, a retrospective study looking at prediagnostic presentations of PD in primary care compared 8,166 PD with 46,755 non-PD cases and found that 5 and 10 years before PD diagnosis, self or general practitioner-reported tremor incidence was significantly higher in those who went on to develop PD (relative risk 7.59).^30^ Reported tremor was therefore a strong predictor of future PD conversion in the general population, whereas iRBD was not, possibly because general physicians and their patients are not wholly aware of iRBD as a diagnostic entity. As an estimated two-thirds of prodromal iRBD participants go on to develop PD or Dementia with Lewy Bodies, we may have detected subtle intermittent postural tremor in iRBD participants with greater sensitivity due to repeated measures using smartphone-based accelerometry. Longitudinal evaluation will enable us to determine whether tremor may subsequently manifest clinically or indeed herald conversion to PD in iRBD participants.

Voice was the most discriminatory factor between iRBD and controls, constituting approximately 50% of the most salient features. These results are consistent with recent studies that have reported vocal abnormalities in individuals with idiopathic iRBD.^17,18^

To assess the reliability and robustness of these findings, a detailed and comprehensive validation of the methodology was undertaken based on (1) evaluation using different CV strategies, (2) analysis of all recordings, along with sex-specific subgroup analyses, (3) classification evaluation using different validation strategies (single and multiple recordings per individual), (4) computation of classification accuracies for different numbers of input features, and (5) identification of the most discriminatory motor tasks between groups. Applying this methodology, we are confident that we have effectively ruled out all major potential confounds in our data set.

Our estimated discrimination accuracies are obtained under realistic, nonlaboratory settings, e.g., in busy outpatient clinic rooms or the home environment. Studies to date have mostly focused on the collection of data in controlled environments under direct supervision.^18,31,32^ In a recent systematic review of new methods for the assessment of PD,^33^ 87% of studies were conducted in a controlled clinic or laboratory setting. To date, those collecting smartphone data in the home environment have typically focused on either upper or lower limb tests of motor function or have incorporated home visits into the study protocol to improve adherence.^34,35^

A reduction in data quality is inevitable when performing studies outside controlled laboratory conditions. Previous studies have reported accuracies of approximately 99% in discriminating PD from controls using features of voice alone based on lab-quality recordings^14^; as anticipated, it was not possible to replicate this same high level of discrimination accuracy here. Instead, integrating features across multiple tasks allows us to offset the reduction in voice data quality and thereby discriminate groups with large effect sizes.

Female participants with iRBD were poorly represented in this study, limiting the inferences it is possible to draw from their inclusion, yet reflecting the established male preponderance in sleep clinic ascertained iRBD cases. Although fewer in number, each female iRBD participant contributed a greater number of recordings compared with their male counterparts (mean 18.5 vs 12.3). However, subgroup analyses of pooled recordings by sex demonstrated effective discrimination accuracies using multiple recordings per participant.

Another potential source of error arises from the possibility of sampling bias within the PD group. Although participants were asked to perform smartphone assessments over a 7-day period at home, up to 4 times a day, it is possible that we captured data from individuals with more optimally controlled PD and/or with assessments less likely to be performed during “OFF” periods. This could have potentially reduced the magnitude of the observed effect sizes that would otherwise be seen. In the absence of independent ground truth data (e.g., video camera and self-reported diary), it was not possible to gauge the level of adherence to the test protocol. For balance and gait tasks, variations in phone placement could have confounded the accelerometer recordings because of multiple known and unknown factors such as pocket size, phone orientation, and phone placement location.

The choice of features extracted in this study was also naturally limited by the single device used for data collection. Studies have used multiple sensors and cameras for data acquisition to compute detailed kinematic gait and balance features.^15^ Using only a single smartphone accelerometer sensor signal, it was not possible to reliably calculate and validate these detailed features. Nevertheless, it is encouraging that even given the fairly free study protocol, differences between groups were sufficient to discriminate with large effect sizes.

One of the potential pitfalls of sophisticated machine learning algorithms including those used in this study^36^ is that high discrimination accuracy does not necessarily translate into high explanatory power. Some of the features we used to help characterize the sensor data were mathematically complex and are not straightforward to interpret from a clinical or etiologic standpoint. The rankings of the most salient features can vary depending on the choice of feature selection algorithm, each scoring the importance of features based on the specific and unique criterion. The sophisticated machine learning algorithms used here can inadvertently make predictions based on factors unrelated to clinical grouping such as factors related to the identity of participants or sex (as opposed to their clinical status), but the evidence rules out these particular confounds being present in this case (data available from Dryad, additional methods, doi.org/10.5061/dryad.3qm0152). Different CV methods may detect such effects to a certain extent, but it is not possible to precisely quantify the influence of all unknown confounders because of the high complexity of these machine learning methods.^37^ Here, we found that LOSO CV discriminatory accuracy was much lower than 10-fold CV (data available from Dryad, table e-9, doi.org/10.5061/dryad.3qm0152), but the mismatch between train and validation set distributions was too large for LOSO CV to be statistically reliable in this case (data available from Dryad, figure e-4, doi.org/10.5061/dryad.3qm0152).

As with all reported studies to date involving the use of wearable technology/smartphones to obtain metrics of disease progression, this study takes the clinician's diagnosis as ground truth, with participants in the PD group judged to have ≥90% probability of PD at their last visit. However, it needs to be remembered that although positive predictive values of up to 99% have been reported in a tertiary specialist movement disorder unit,^38^ accuracies vary, and up to 25% of individuals diagnosed with PD in life may be found to have an alternative diagnosis at death should they proceed to neuropathologic examination.^39^ It is with this in mind that the importance of studies involving the follow-through of deeply phenotyped participants with iRBD and PD to autopsy cannot be underestimated to fully realize the potential of objective data from wearable sensors.

Our initial findings are highly promising, but further refinement of the algorithms is required in terms of external replication. In addition, it would be valuable to include other parkinsonian and tremulous conditions as comparators, which may be more difficult to differentiate from PD. Therefore, from a clinical perspective, we may have overestimated the diagnostic utilities of our extracted features because of “spectrum bias,” although this is less of an issue for the iRBD control comparison. We also aim to derive quantitative measures that can be compared against clinician-assigned measures of disease severity, namely the MDS-UPDRS to monitor disease progression. Home-based testing would allow a more comprehensive assessment of a participant's condition through repeated measures over several days, as opposed to the current reliance on infrequent and single time point assessments captured in clinic, which are subject to many confounds. The use of fully automated methodology, which we intend to make entirely open access, will further facilitate replication and should make this an ideal marker for use in clinical trials.

Objective measures of motor symptom severity would also be of direct benefit in optimizing treatment strategies in complex disease, including the use of apomorphine, deep brain stimulation, and Duodopa therapies. This would empower people with PD to effectively self-manage their symptoms remotely at home, allowing titrations to be made directly according to medication response. Such a collaborative approach to long-term disease management may help to address the increasing demands on health care services from an aging population at an ever-increasing risk of neurodegenerative diseases including Parkinson disease.

This study uniquely demonstrates the use of consumer-grade smartphones to capture real-world data capable of distinguishing a large number of 522 PD, iRBD, and control participants with 84.6%–91.9% sensitivity and 88.3%–90.1% specificity. We continue to work toward the ultimate goal of developing the tools to allow the reliable and sensitive quantification of changes in disease severity over time, thereby facilitating individual-level stratification of prodromal and early PD participants to allow the identification of at-risk individuals and track response to future critically needed neuroprotective therapies.

## Publication history

Received by *Neurology* April 30, 2018. Accepted in final form July 12, 2018.

## Glossary

CV: cross-validation
IMU: inertial measurement unit
iRBD: idiopathic REM sleep behavior disorder
LOO: leave-one-(recording)-out
LOSO: leave-one-subject-out
MDS: Movement Disorder Society
MDS-UPDRS: Movement Disorders Society-Unified Parkinson's Disease Rating Scale
OPDC: Oxford Parkinson's Disease Centre
PD: Parkinson disease
UPDRS: Unified Parkinson's Disease Rating Scale
